# Genetics in ophthalmology: molecular blueprints of retinoblastoma

**DOI:** 10.1186/s40246-023-00529-w

**Published:** 2023-09-01

**Authors:** Leon Marković, Anja Bukovac, Ana Maria Varošanec, Nika Šlaus, Nives Pećina-Šlaus

**Affiliations:** 1 Department of Ophthalmology, Reference Center of the Ministry of Health of the Republic of Croatia for Pediatric Ophthalmology and Strabismus, University Hospital “Sveti Duh”, Zagreb, Croatia; 2 Faculty of Dental Medicine and Health Osijek, Josip Juraj Strossmayer University of Osijek, Osijek, Croatia; 3 Department of Biology, School of Medicine, University of Zagreb, Šalata 3, 10000 Zagreb, Croatia; 4 Laboratory of Neurooncology, Croatian Institute for Brain Research, School of Medicine, University of Zagreb, Salata 12, 10000 Zagreb, Croatia

**Keywords:** Retinoblastoma, RB1, Genetic testing, N-myc

## Abstract

This review presents current knowledge on the molecular biology of retinoblastoma (RB). Retinoblastoma is an intraocular tumor with hereditary and sporadic forms. 8,000 new cases of this ocular malignancy of the developing retina are diagnosed each year worldwide. The major gene responsible for retinoblastoma is *RB1*, and it harbors a large spectrum of pathogenic variants. Tumorigenesis begins with mutations that cause *RB1* biallelic inactivation preventing the production of functional pRB proteins. Depending on the type of mutation the penetrance of RB is different. However, in small percent of tumors additional genes may be required, such as *MYCN*, *BCOR* and *CREBBP*. Additionally, epigenetic changes contribute to the progression of retinoblastoma as well. Besides its role in the cell cycle, pRB plays many additional roles, it regulates the nucleosome structure, participates in apoptosis, DNA replication, cellular senescence, differentiation, DNA repair and angiogenesis. Notably, pRB has an important role as a modulator of chromatin remodeling. In recent years high-throughput techniques are becoming essential for credible biomarker identification and patient management improvement. In spite of remarkable advances in retinoblastoma therapy, primarily in high-income countries, our understanding of retinoblastoma and its specific genetics still needs further clarification in order to predict the course of this disease and improve therapy. One such approach is the tumor free DNA that can be obtained from the anterior segment of the eye and be useful in diagnostics and prognostics.

## Background

The field of ophthalmology genetics and genomics is expanding fast and the accumulated knowledge aims to develop novel and improved diagnostic and therapeutic approaches. The specific molecular mechanisms behind eye diseases, including retinoblastoma (RB), are being recognized every day, because it is important to understand their genetic causes and biological behavior in order to improve clinical outcome. Genetic implications for more than 97% of all RB cases is the *RB1* gene inactivation. Although retinoblastoma has been genetically characterized a long time ago [1], its molecular blueprint is still incomplete and needs deeper investigation. The foundational work that explained the retinoblastoma inheritance but also the concept of tumor suppressor genes was originally published by Knudson in his seminal paper from 1971 [2]. Knudson’s two hit model proposed that one *RB1* allele is lost or mutated in all cells and a second somatic mutagenic event affects the remaining allele in a primitive retinal cell, thus initiating tumorigenesis. More than 10 years after Knudson’s discovery, *RB1* gene was the first tumor suppressor gene to be identified and cloned [3–6].

While there have been remarkable advances in retinoblastoma therapy over the past decades, such as intra-arterial, intraocular chemotherapy or proton-beam radiation therapy, at present there remains a huge treatment option bias between high, and low- and middle-income countries. Treatment options are chosen, among other things, on the basis of the intraocular classification of retinoblastoma (the Intraocular Classification of Retinoblastoma ICRB) [7]. Several classification systems are currently in use, for example, the IIRC (International Intraocular Retinoblastoma Classification), ICRB and cTNMH (American Joint Committee on Cancer (AJCC)) and for extraocular disease, the IRSS (International Retinoblastoma Staging System) and cTNMH staging [8].

In this review we bring an overview of retinoblastoma genetics, inheritance mechanisms that are behind the development of retinoblastoma. We also discuss the structure and molecular functions of *RB1* gene and its protein, consequent clinical behavior it influences, as well as current therapeutic approaches.

## Retinoblastoma

Retinoblastoma is an aggressive rare childhood cancer of the developing retina that is initiated by biallelic *RB1* gene inactivation. Although rare, it is the most common primary intraocular tumor in children and infants with the approximate incidence of 1/15–20,000 live births [9–11]. This genetic disease (OMIM 180200) can present with bilateral tumor involving both eyes, or unilateral tumor involving only one eye. The mean age of diagnosis is 12 months for bilateral and 24 months for unilateral.

All neuronal cell types of mammalian retina are derived from a common retinal progenitor cell (RPC). At specific stage of development RPC can give rise to precursor cells, that have the potential for terminal differentiation into a specific subset of retinal cell types [12]. Several papers debated on the cell of origin of retinoblastoma. However, novel research establishes [13, 14] that the cell-of-origin of human retinoblastoma is a cone photoreceptor precursor. This neuronal cell type is uniquely susceptible to malignant transformation since it remains within the inner nuclear layer of the infant retina. Losing both alleles of the *RB1* tumor suppressor gene in such susceptible developing retinal cell initiates the benign precursor, retinoma, which usually progresses to retinoblastoma with accumulation of increasing genomic changes and uncontrolled cellular proliferation [13–15].

Mixed genetic profiles of retinoblastoma cells that hold molecular characteristics of retinal progenitors but also other cell types including cone photoreceptor precursor, indicated the possibility of existence of different molecular subtypes of retinoblastoma [12, 16]. Based on precursor cells a multi-omics study by Liu et al. identified two molecular subtypes of retinoblastoma, cone-like and cone/neuronal. Most of the heritable forms expresses mature cone markers and show less genetic alterations besides *RB1* inactivation, while cone/neuronal express markers of less differentiated cone together with neuronal/ganglion cell markers, and are associated with stemness. This subtype also shows frequent recurrent genetic alterations including *MYCN*-amplification. This study indicates important biological and clinical perspectives for retinoblastomas diagnosis and prognosis [16, 17].

## Mutational characteristics of inherited and sporadic retinoblastoma

**Fig. 1:**
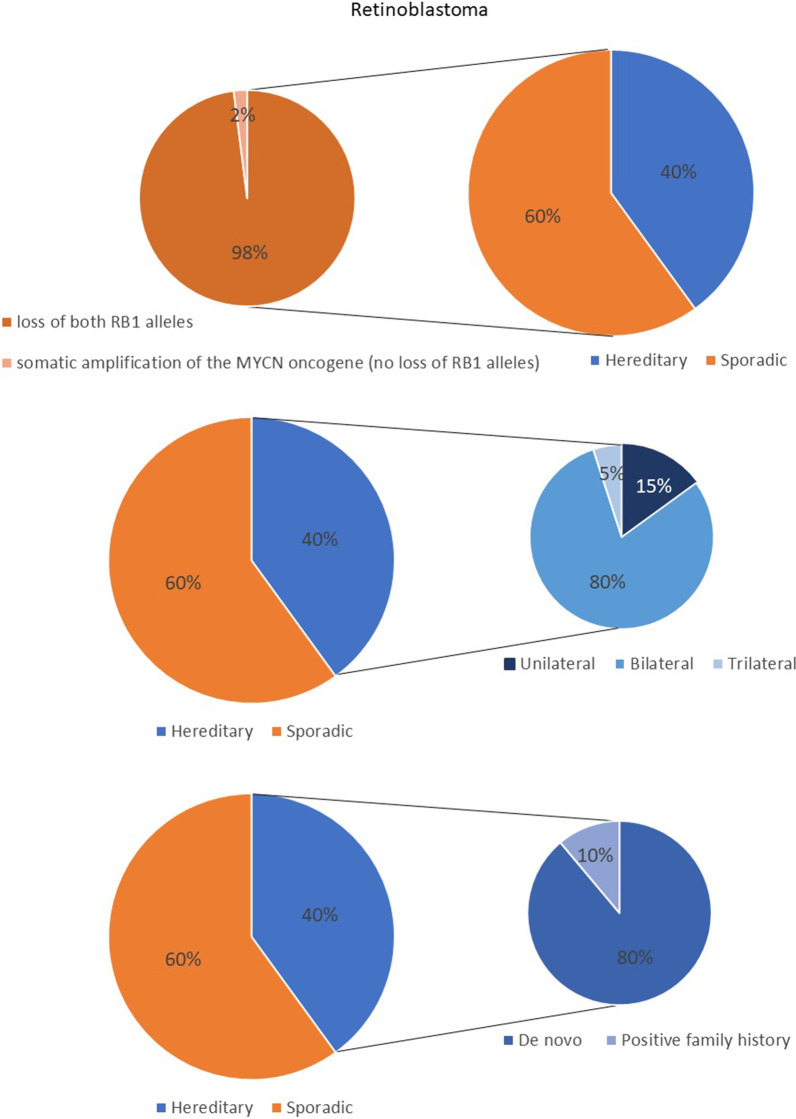
Visual representation of two major genetic forms of retinoblastoma: hereditary (also known as germline) and non-hereditary (sporadic or somatic)

There are two major genetic forms of retinoblastoma: hereditary (also known as germline) and non-hereditary (sporadic or somatic). In the hereditary form of retinoblastoma one mutation of *RB1* gene already exists in the genome of the zygote and will be present in every cell of the body, so there is a higher risk of sustaining secondary somatic mutation in the retina and the consequent development of retinoblastoma tumor. Hereditary retinoblastoma accounts for 40% of all cases; 80% of them are bilateral, 15% unilateral, and 5% trilateral (bilateral retinoblastoma with pineal/midline neuroectodermal tumor) [13, 16, 18, 19] (Fig. 1).

Patients with bilateral or multiple tumors can be presumed to have a germline pathogenic *RB1* mutation which can be passed to their offspring. Besides, germline mutations in hereditary form predispose patients at a lifelong risk of developing other types of tumors, ocular and non-ocular [16]. People with hereditary retinoblastoma may have a family history of the disease; however, many mutations can also arise de novo during embryonic development [20]. In hereditary retinoblastoma only 6–10% of patients have a positive family history of retinoblastoma, while those with negative family history can sustain up to 80% of de novo mutations [21]. All the bilateral cases appear to be hereditary; however, 80% of them occur because of de novo mutations since neither parent was affected. However, around 15% of the unilateral cases can also be hereditary [18, 21, 22]. Li et al. [23] aimed to determine the etiological role of de novo mutations in different inherited eye disease and showed that among them retinoblastoma was the disease with the highest incidence of de novo mutations.

The mode of inheritance of germline retinoblastoma is autosomal dominant. This means that in order for retinoblastoma to develop, one copy of mutated *RB1* gene needs to be inherited from one parent while the other copy, inherited from the other parent, has to be struck by mutation in retinal cells. The altered gene may be the result of a new mutation (mutation de novo) that occurs in an egg or sperm cell or just after fertilization. The second mutation usually occurs in childhood, frequently leading to the development of bilateral retinoblastoma.

The other 60% of retinoblastomas are non-hereditary or sporadic (Fig. 1). The majority of patients present with unilateral tumor and have a somatic pathogenic *RB1* mutation. 98% of non-hereditary retinoblastoma result from somatic loss of both *RB1* alleles in a retinal precursor cell [13]. The remaining 2% of non-hereditary tumors are formed by somatic amplification of the *MYCN* oncogene while their *RB1* alleles remain normal. These tumors are always unilateral, and diagnosed at median 4.5 months. Somatic mutations cannot be passed to the next generation since they are present only in cells of the tumor tissue, and not in all body cells including egg or sperm cells. Here there is no family history of the disease. Sporadic cases are born with two normal copies of the *RB1* gene. However, the sporadic cases arise from two spontaneous mutations affecting both alleles. This happens in the same somatic retinal cell usually in infancy. Notably, it can be challenging to determine if a patient with unilateral retinoblastoma has hereditary or sporadic form. For this situation genetic testing is indicated.

Another type of inheritance needed mentioning is mosaicism. Mosaicism can be defined as the presence of two or more genetically separate sets of cells in a person’s (patient’s) body. This usually happens after the zygote is formed and such embryonal mutations result in constitutional mosaicism. Although, mosaicism in an unaffected parent of a sporadic Rb patient is very rare around 0.7% [24–26], it is important to determine whether the mosaic pathogenic *RB1* mutation happened before the zygote is formed (preconception mutation) or after it is formed (postconception mutation). If the pathogenic mutation is a postzygotic event this is a somatic mosaicism and affects only a portion of the body. Notably, based on cellular developmental stages, whether the mutation occurs before or after in cells that are going to develop into gonads, somatic mosaicism may or may not involve germ cells. The pre-zygotic mutational event passed on by a mosaic parent that himself does not have retinoblastoma implies risk in future offspring and the genetic testing is recommended.

At the cellular level, the inactivation of one *RB1* allele in heterozygous state develops a normal phenotype. In hereditary retinoblastoma, offspring have a 50% chance of inheriting the mutant *RB1* allele. However, retinoblastoma phenotypically presents in an autosomal dominant manner with 90% penetrance. Penetrance of the RB phenotype is ultimately based on RB1 protein expression, which, in turn, is dependent on the type and nature of the underlying genetic mutations. A low penetrance RB phenotype is associated with in-frame variants, missense or known splice site variants, and indels in exon 1 or the promoter region. On the contrary, null mutations and nonsense variants typically demonstrate complete penetrance of the RB phenotype. So, the gene, although mutated, can be non-penetrant and the patient does not express the disease. In other words, genotype–phenotype correlation is important for clinical presentation of the disease. [27, 28]. In addition, it has been shown that the difference in clinical manifestation is also linked to the so-called parent-of-origin effect where parental origin of the pathogenic allele is responsible for the penetrance. It has been demonstrated that when the variant is inherited from the father [29] a higher penetrance of the mutated gene is observed.

## *RB1* gene and mutational spectrum

The *RB1* gene is located on chromosome 13q14.2, spans 180 kb and consists of 27 exons [30]. The gene is also called RB Transcriptional Corepressor 1 and it encodes an mRNA transcript of 4.7 kb. To be more precise the gene spans from chr13:48,303,744 to 48,599,436 (GRCh38:CM000675.2) (GRCh38/hg38) according to databases Ensemble and Genecards and encompasses 295,693 bases (https://www.genecards.org/cgi-bin/carddisp.pl?gene=RB1) and holds a core promoter.

**Fig. 2:**
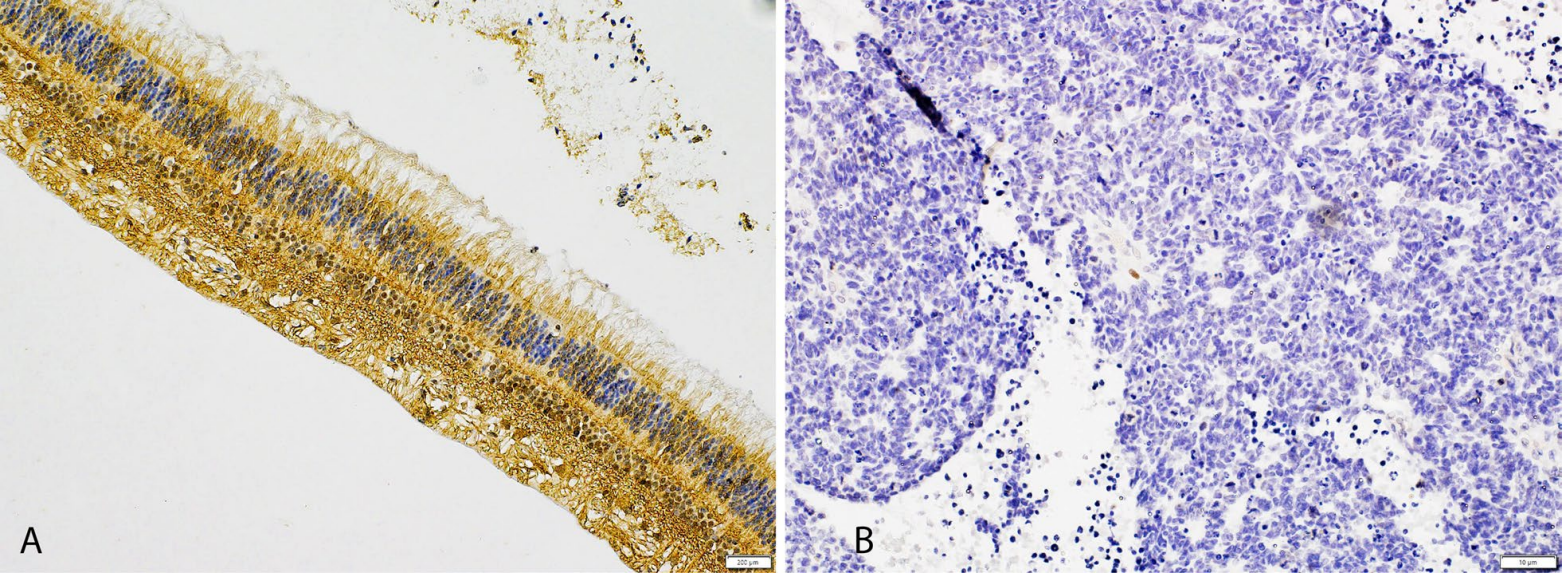
Representative images of pRB immunohistochemical staining. **A** Intense and diffuse expression in a large majority of normal retinal cells. **B** absence of expression in tumor cells. Scale bar is 10 µm for **A**, and 200 µm for **B**

Retinoblastoma gene harbors a large spectrum of pathogenic variants, around 2500 discovered so far, with more than 500 different somatic or germline mutations resulting in *RB1* inactivation (RetNet; https://sph.uth.edu/retnet/). Biallelic inactivation of the *RB1* gene is achieved by mutations in both *RB1* alleles or more commonly due to loss of heterozygosity (allele gross deletions). All things considered, most mutations in the *RB1* gene prevent it from making any functional protein, so cells are unable to regulate cell division effectively (Fig. 2).

The majority of pathogenic point mutations are known to be distributed within exons 1–25. Eighty-five percent of them are single nucleotide variants (SNV) or insertion-deletions (indels) that lie within the 180 kb of the RB1 coding sequence, while some changes were mapped onto the promoter or introns [16, 31, 32]. Recently many modern high-throughput techniques identified novel pathogenic variants of *RB1* gene. The mutational pathogenic spectrum primarily includes nonsense mutations that introduce a premature stop codon. Out-of-frame exon skipping due to splice site variants are also commonly found resulting in truncated proteins as well. Splice donor site mutations at the CGA codon of intron 12 have also been reported. Intronic variants, highly polymorphic microsatellites (Rb1.20), and minisatellites have further been reported to disrupt *RB1* gene functioning [33, 34].

Besides point mutations, other types of alterations are also common, including chromosomal rearrangements, large exonic deletions, and the aberrant methylation of the gene promoter region, [16, 18, 31, 32, 35].

The investigation using mutational screening by Price et al. [32], identified substitutions as the most common mutation type (58.4%), followed by small length mutations (22.9%), and large deletions (12%). The majority of mutations introduced stop codons (58.2%) followed by splicing anomalies (19.2%), while missense mutations were in the minority (3.5%). This study also reported on frequent recurrent mutations and also identified mutational hotspots. Hotspots with mutation rates of 58.6% were located to the regions coding pocket domains of the RB1 protein, namely pocket A, codons 379–578 (exons 12–18), pocket B, codons 645–787 (exons 19–23) and spacer region, codons 579–644 (exons 18–19). These findings were confirmed by Tomar et al. [36] who also observed a high mutation rate in the exons coding for pocket domains in 58.1% of patients using a combination of approaches including Multiplex Ligation-dependent Probe Amplification (MLPA) assay, deletion screening, direct sequencing, copy number gene dosage analysis and methylation assays. As far as recurrent mutations are in question, both studies report on relatively high percentage (about 44.3%) of recurring point mutations. Important to note is that the most frequent recurring mutation was p.Arg320* found in exon 10, followed by p.Arg358* (Exon 11), p.Arg455* (Exon 14), p.Arg445* (Exon 14) and p.Tyr498* (Exon 16) variants. Mutations p.Arg579Glnfs*29 (Exon 18), p.Arg787* (Exon 23), p.Arg255* (Exon 8) and p.Arg552* (Exon 17) were also found each in two patients.

*RB1* gene is known for its CpG islands dispersed across several exons—exon 8, 10, 11, 14, 15, 17, 18 and 23, and these locations represent mutational hotspots for recurrent mutations. Point mutations in these 8 exons have been reported previously in Chinese population at variable frequencies [36, 37].

The detailed *RB1* mutational signature identified in the above-mentioned study by Tomar et al. [36] on 50 retinoblastoma patients from Singapore displayed a total of 61 *RB1* germline and somatic point mutations. The prediction of the severity of mutations was also investigated by four in silico analyzes tools—PROVEAN, Mutation Taster, SIFT and CADD. The incidence of mutations showed nonsense mutations (stop codon gained) occurring at 55.7%, followed by 24.6% frameshift, 9.8% splicing, 8.2% missense and 1.64% promoter alterations. The majority of point mutations, precisely 57.4% were confined to exons coding for pocket domains of pRB, involved in regulation of transcription [36].

**Fig. 3:**
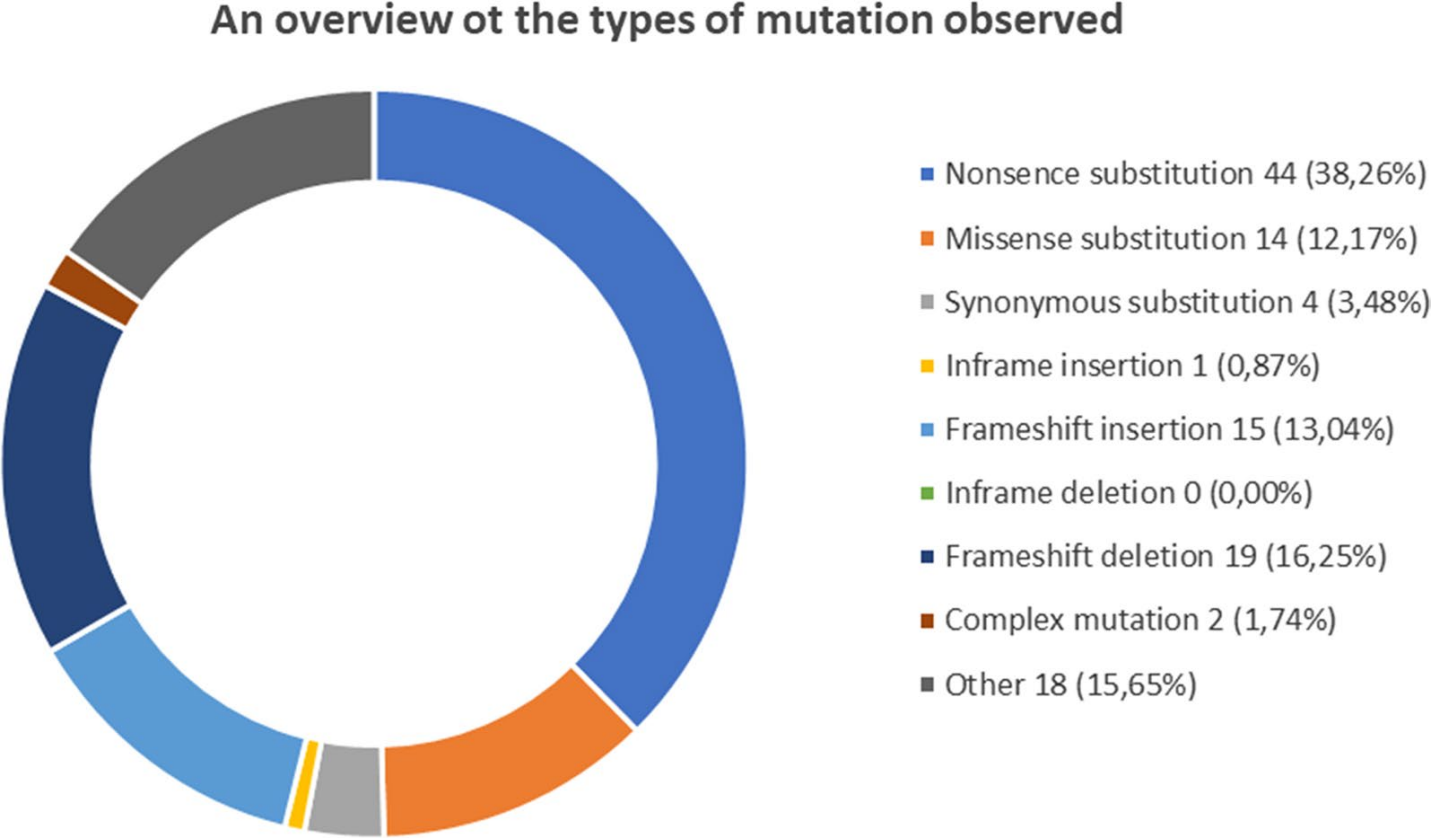
An overview of the types of mutations observed in retinoblastoma. Adapted from COSMIC database (https://cancer.sanger.ac.uk/cosmic/browse/tissue?hn=retinoblastoma&in=t&sn=eye&ss=all)

Another study [27] that investigated *RB1* mutation types showed that the most common mutations were stop codon gained (38.2%), splicing error (19.7%) and large deletion (15.8%). Small deletions 11.8%, small insertion, 7.9% and missense variants 5.3% were found to be less frequent. Similar types of mutations and frequencies are also reported and can be found in COSMIC database as shown in Fig. 3.

It is important to highlight that depending on the type of mutation the penetrance of RB is going to be different. In general, mutations that generate early stop codons are associated with high penetrance RB. However, some other of the identified mutations were associated with incomplete penetrance of the disease. These include missense mutations, the most common of which was in exon 20 (p.R661W). [32]. Other low penetrance mutations were attributed to missense changes leading to splicing alterations, promoter mutations, in-frame deletions, and splice consensus alterations. Additionally, some exon 1 null mutations were associated with reduced expressivity, late onset and incomplete penetrance as reported previously [38, 39].

## pRB protein structure and function

Human RB protein (pRB) has a mass of 106 kDa (106,159 Da) and consists of 928 amino acids. It has been demonstrated that this protein functions in a wide network of molecular interactions [3, 18]. The function and the structure of this protein has been extensively studied for decades; however, its many molecular functions still need investigation. Roughly the pRB consists of three domains: the N-terminal domain (RBN), a small “pocket” structure with A and B pockets also known as the A/B region (RBP), and the C-terminal domain (RBC). An evolutionarily conserved spacer region of 71 amino acids separates the A and B domains and is necessary for the formation of the pocket. Specific site LxCxE exists with which pRB binds to chromatin remodeling proteins BRG and histone deacetylases (HDACs). The two domains that form the AB pocket are critical for tumor suppressor activity [30], while the C-terminal region complexes with E2F transcription factor causing cell growth arrest. The N-terminal region besides containing CDK phosphorylation sites, is additionally involved in receptor-targeted chromatin remodeling.

The pRB protein is involved in the cell cycle as a regulator of cell division. Its main function is in G1 phase, where the unphosphorylated (or hypophosphorylated) pRB participates in regulation by binding to E2F transcription factor, which ultimately results in cell cycle arrest. After binding to pRB, E2F cannot carry out the transition of the cell through the G1 to the S phase. The complexes of cyclin and cyclin dependent kinase (CDK) phosphorylate pRB and such phosphorylated retinoblastoma protein dissociates from E2F. Released E2F facilitates transcription of E2F-responsive genes necessary for cell cycle progression. Therefore, when negative regulatory function is lost, such as in the setting of retinoblastoma, abnormal progression through the cell cycle due to constant activity of E2F can lead to uncontrolled proliferation and tumor development.

In mammalian cells signaling to the RB pathway and G1 control is achieved mainly through cyclins, CDKs and CDK inhibitors (CKIs). The signal starts by the synthesis of D-type cyclins that activate CDK4 and CDK6. Further mitogenic signals induce E-type cyclins to activate CDK2. These two cyclin/CDK complexes, cyclin D and CDK4/6 and cyclin E and CDK2 cooperatively phosphorylate RB-family proteins. Such phosphorylation derepresses E2F and the transcription of E2F-target genes that promote G1/S transition is allowed [40]. CDK4 and CDK6 are inhibited by INK4 proteins (CDKN2A or p16), whereas the p21 (CIP/KIP) family of CKIs inhibits multiple CDKs. The regulation of both G1 cyclins and CDK inhibitors is evolutionarily conserved.

The role of pRB in cell cycle is best studied; however, many cellular signaling pathways are linked to G1 phase, a phase controlled by RB signaling pathway [1], so pRB has many additional functions. Knowledge brought by the global transcriptomic and methylation profiles of retinoblastoma cells greatly improved the understanding of the biology of this tumor and pRB protein. pRB protein versatile functions are illustrated by many different protein interactions it performs. Besides E2F family members, pRB interacts with chromatin modifiers such as DNMT1, HDAC, SIRT1, the replication factor C, the DNA polymerase α, MDMX and MDM2 [3]. For example, it has been shown that MDM2 is an important regulator of the RB activity with dual roles, when in G1 phase it can bind to the RB mRNA and enhance the RB translation, while during G2 MDM2 can degrade pRB.

It has also been shown that pRB protein stabilizes constitutive heterochromatin to maintain the overall chromatin structure [41, 42]. Diverse chromatin remodeling enzymes can bind to the LxCxE conserved domain of pRB protein. Therefore, this domain is necessary to modulate transcriptional activation by histone methylation and nucleosome remodeling [43]. Dephosphorylated pRB calls on chromatin remodeling proteins HDAC and BRG to the RB-E2F complex and causes chromatin structure alteration. In such a fashion, the access of transcriptional machinery is prevented and the transcription is suppressed. Another molecule with which pRB1 can interact is Suv39h1 histone methyltransferase [3] which functions together with the heterochromatin proteins (HP1).

The pRB1 effect on chromatin structure also implicates this protein in mitosis, as decondensation and condensation of chromatin are main features of karyokinesis. Growing evidence reveals a crucial role of pRB1 in modulating chromatin structure and chromosome segregation. Moreover, it was shown that RB interacts with the mitotic spindle organizer NuMA. Emerging knowledge shows that when the pRB1 protein is non-functional this can lead to chromosomal instability (CIN) [44] characteristic for tumor cells.

Knowledge of the pRB additional functions is continuously increasing. Today we know that pRB is involved in DNA replication inhibition, apoptosis, cellular senescence, differentiation, DNA repair and angiogenesis. [45]. Depending on cellular contexts pBR1 role in apoptosis is dual, it can either stimulate the apoptosis process or promote anti-apoptotic factors. Under stress or hyperproliferation pRB1 plays an important role in blocking apoptosis in an RB-E2F1-dependent fashion [1, 3]. In these circumstances pRB1 is hyperphosphorylated and changes structure to release E2F. Accordingly, when pRB1 is lost apoptosis is induced. Cellular senescence is yet another event controlled by RB proteins [11, 46]. The molecular explanation of all the above-mentioned cellular processes is beyond the scope of this paper and can be found in two critical reviews by Dick and Rubin [47] and Indovina and coauthors [45].

## Other oncogenic mutations in Retinoblastoma

While the initiation of tumorigenesis in RB begins with mutations in the *RB1* tumor suppressor gene causing biallelic inactivation, additional genetic and genomic arrangements may be required for continued growth and progression. Driver mutations give advantage of selective growth while passenger contribute to progression events. However, the known mutational landscape in RB is still rudimentary. Additional genetic causes of RB are primarily confined to *MYCN* gene. However, they are usually mutually exclusive to *RB1* mutations. Amplification and increased expression of this gene represent genetic blueprint in a small frequency (< 2%) of rare and aggressive subset of non-hereditary retinoblastomas. These tumors are initiated by high *MYCN*-amplification without *RB1* inactivation [48]. Furthermore, recurrent mutations in *BCOR* and *CREBBP* genes have been described in a small percentage of tumors.

Karyotype analyses and comparative genomic hybridization (CGH) revealed that another common mechanism for tumorigenesis in RB is somatic copy number alterations (SCNAs). Several highly recurrent alterations were identified from tumor studies such as gains of 1q, 2p, 6p, and losses of 13q and 16q14 [49]. The gain of 6p is particularly common in RB tumors and has been associated with significantly lower rates of ocular salvage. CNA also included deletions of the following tumor suppressor genes that might contribute to retinoblastoma tumorigenesis: *TP53, CDH13, GATA5, CHFR, TP73* and *IGSF4*. [50]

The relevant genes residing in those loci have been found to also show alterations in their copy number. Thus gains (4‒10 copies) in oncogenes *MDM4, KIF14* residing on 1q32, followed by gains of *MYCN* on 2p24 and *DEK* and *E2F3* on 6p22 have been observed, while loss of the tumor suppressor gene *CDH11* was found on 16q22-24 [13]. Other recurring changes include *OTX2* amplification and *BCOR* mutations or loss [16], but they occur in a small minority of retinoblastomas. Examples of other candidate genes are *RBL2, CREBP, MGMT, RASSF1A, CASP8* and *MLH1.*

Notably some of the above listed genes have been shown as good diagnostic and predictive biomarkers. Madhavan et al. [51] validated the overexpression of *KIF14* and *E2F3* and showed that both are associated with tumor progression [52].

Biomarkers can be divided in those assessed by invasive methods sampled from tumors, and noninvasive coming from the specific sample sources like blood, plasma, serum and aqueous humor. Proposed additional biomarkers include APOA1 (Apolipoprotein A1), CDKN2A (p16INK4A), CRABPs (Cellular Retinoic Acid Binding Proteins), GFAP, RBP3, LMNB1 (Lamin B1), TFRC (Transferrin Receptor), SOX2, Survivin, MDM2, MDMX, NOL7 [3, 53, 54].

Of note is that retinoblastoma presents with all categories of aberrant splicing. The most predominant splicing events are exon exclusion and mutually exclusive exons. Furthermore, these differential splicing events enriched the activity of E2F family transcription factors, the visual sense gene ABCA4, and the skysplicing factor DAZAP1. One other well-known event in retinoblastoma occurs in MDM4, where exclusion of exons 6 or 9 (skipping of exons 6 or 9) produces the oncogenic isoforms. Additionally, the Dab1 and RB1 genes undergo both exon inclusion and exclusion events, and various exon skipping events [55].

Recently, metabolomic and integrated omics (ATAC/RNA seq) studies have identified unique biomarkers in retinoblastoma. Studies uncovered unique functional associations between genes and metabolites by integrating transcriptomic profiling derived from tumor tissues and metabolomics from tumorous eye vitreous humor samples [56, 57]. Performing the single cell RNA- and ATAC-Seq analyses of primary tumor tissues, predominant presence of cone precursors at different stages of the cell cycle in the Rb tumors have been revealed [58]. Global metabolomic and integrated omics analysis of the tumorous eye tissue and VH revealed uniquely altered biomarkers, indicating diversion of the tumor metabolism from healthy retina, providing a database regarding aqueous humor and tumor tissue related to RB disease that may be used as a source of biomarkers (56–58]. Protein biomarkers were assessed by comparative parametric analyses [56] that revealed additional proteins expressed in the retinoblastoma that were not expressed in the control group. These were histone H2B type 2-E (HISTH2B2E), InaD-like protein (PATJ) and ubiquitin conjugating enzyme E2 V1 (UBE2V1). OpenTarget Tool software indicated that glyceraldehyde 3-phosphate dehydrogenase (GAPDH) and CD44 were also more highly expressed in the retinoblastoma. Babu et al. [57] identified distinct dysregulated gene clusters, that were considerably different between retinoblastomas and the controls. Genes that were identified as upregulated comprised *E2F1, E2F2, CCNB2, CCNE2, CDK1, CDKN2A* and *CHEK2*. Furthermore, the significant upregulation of immune system-related genes such as *CD86 *and *CD19*, and epigenetic regulators such as SYK and PRDM1 was also found. Mitochondrial TCA-related *FAHD1* gene was also significantly upregulated in retinoblastoma patients. On the other hand, genes that were downregulated comprised photoreceptor-related genes such as *RHO, NRL, PDE6D, CRABP1,* glycolytic factors such as *HK1, SLC2A1 *and *FOXO3*, as well as methyltransferase *MGMT* and *RB1*.

NGS technologies have also paved the way for investigating the role of non-coding RNA, such as miRNAs and long non-coding RNA (lncRNA), in RB development. The transcriptional, post-transcriptional and epigenetic regulatory functions of lncRNAs can either be oncogenic or tumor suppressive [59].

Through NGS methods, several lncRNAs have been identified as differentially expressed in RB and as potent regulators of RB progression and metastasis, including *BANCR, AFAP1-AS1, NEAT1, XIST, ANRIL, PlncRNA-1, HOTAIR, PANDAR, DANCR* and *THOR*.

Various studies have investigated the involvement of miRNAs too and demonstrated that some are downregulated in retinoblastoma, while others were upregulated. miRNAs found to be downregulated are miR-216a, miR-217, let-7a, let-7i, let-7f, miR-9, miR-92a, miR-99b, while those found to be upregulated are miR-103, miR-142-5b, miR-106b, miR-143, miR-148b, miR-17, miR-16, miR-183, miR-182, miR-19a, miR-18a, miR-29a, miR-29b, miR-29c, miR-20a, miR-30b, miR-30d, miR-34a, miR-494, miR-378, miR-513, miR-513–1, miR-513–2, miR-518c, miR-96. Furthermore, Liu et al. [17] demonstrated that miR-320, let-7e and miR-21 were dysregulated in plasma of Rb patients.

Yang and Mei [60] established differentially expressed miRNA signatures during retinoblastoma progression and found 14 mirRNAs,—miR-20a, miR-373, miR-125b, let7a, let-7b, let-7c, miR-25 and miR-18a. Another in silico study by Venkatesan et al. [61] showed miR486-3p and miR-532 to be downregulated too. miR-3613 has also been established as important since it can target more than 36 tumor suppressor genes. There is also a group of miRNAs that are specifically regulated under hypoxic conditions and are called hypoxia-regulated microRNAs. Such miRNAs have been found in retinoblastoma too, with most important ones miR181b, miR30c-2, miR125a3p, miR497 and miR491-3p [59].

## Epigenetic mechanisms

Besides involvement of non-coding RNAs described above, other epigenetic mechanisms have also been found to be causative for retinoblastoma etiology. Global methylation profiles of retinoblastoma cells provided novel insights that epigenetic changes are required to promote retinoblastoma growth and progression. Several studies showed that epigenetic deregulation of tumor-promoting pathways is important for RB formation and progression beyond *RB1* inactivation. This indicates that epigenetic blueprint could also serve as predictive molecular biomarker. However, the prognostic value of retinoblastoma DNA methylation profile has only been proposed recently [62, 63].

Retinoblastoma includes alterations that result from aberrant methylation of the promoter of many relevant genes. Needless to say, one of the first genes investigated for DNA methylation was *RB1* gene. The 5’ region of the *RB1* gene contains a CpG island that encompasses its essential promoter region that is normally unmethylated. *RB1* promoter DNA hypermethylation is a known cause of sporadic retinoblastoma and correlates with decreased expression of pRB1 protein [64]. Hypermethylation of the *RB1* gene occurs in 13% of sporadic unilateral tumors and it was also reported that paternal allele was specifically methylated [65]. Li and coauthors [63] performed genome-scale DNA methylation profiling in order to identify the methylation status of retinoblastoma tumors. They found 294 genes that were directly regulated by promoter or gene body DNA methylation. Studies on retinoblastoma have inquired into the methylation status of other genes as well. Some of the genes with aberrant methylation of promoters were identified such as *MGMT*, *RASSF1A*, *CASP8*, *MLH1* genes [66, 67], *CDKN2A/p16INK4A, VHL, LDHA, RUNX3, APC2, PAX5* and many others [65]. For instance, *RASSF1A* was hypermethylated in 59% of RBs tumors, *APC* in 6%, while *MGMT* in 15% of the retinoblastomas analyzed. We also have to mention a histone methyltransferase, an enzyme that is specific for retinoblastoma and is found to be overexpressed in tumors. This is enhancer of zeste 2 polycomb repressive complex 2 subunit gene (*EZH2*).

The methylation status of promoters and the accompanying somatic mutational changes both define the aggressiveness of retinoblastoma.

Different omics technologies, revealed additional genes involved in retinoblastoma etiology [68]. Using single-cell RNA sequencing, the expression levels of UBE2C—an oncogene that promotes growth and proliferation, were found remarkably higher in retinoblastoma tissues of metastatic patients [69]. Next, genes found upregulated and overexpressed in retinoblastoma are *NEK7* [28] and *SKP2*, while *ARHGAP9* and *DRAIC* expression levels [52] were significantly decreased as shown by RNA-sequencing analysis. Of note is that the decreased levels of ARHGAP9 were found to significantly affect susceptibility toward chemotherapeutic drugs. Whole exome sequencing, somatic copy number alterations profiling, microarray analyses, collectively, revealed additional candidates, *DEK, CRB1*, *MIR181*, *NUP205*, *IL8*, *IL6*, *MYC* and *SMAD3* [70–73], overexpressed in retinoblastoma and usually associated with aggressive retinoblastomas[69]. Integrated Mutation Profiling of Actionable Cancer Targets, a tumor-profiling test developed by Memorial Sloan Kettering revealed downregulated genes namely *CREBBP, HIST1H4H*, *RELN*, *RPTOR*, *TERT*, *MSH3*, *TSC2*, *ARID1A*, *CDK4*, *BRAF*, *JAK1* and *ROCK1* [74, 75]. Another approach—proteomic profiling of retinoblastoma tumors was also performed which offered additional relevant protein products involved. Upregulation of IGF2BP1, SOX4 and B7H3 proteins was observed. Furthermore, chromogranin A, Rac GTPase-activating protein 1, fetuin A, midkine, LRP1, COMP, TGB3, TLN, FLNA, OGN, A1BG, Serpin A1, ORM2, LRG1, CHI3L1, apolipoprotein A1, transferrin, alpha-crystalline A and CRABP2 proteins have been found to be upregulated too [76–78]. Contrary, mass spectrometry showed that levels of proteins PEDF and nucleolin (NCL) were downregulated in retinoblastoma.

Epigenetics studies identified SKY protein levels to be epigenetically upregulated, furthermore high expression of DNMT1a and DNMT3a proteins were also identified to be particularly correlated with malignant phenotypes. HELLS protein was downregulated, while PLK1 was upregulated [52]. Similarly, epigenetic silencing of *MGMT*, *CDK1, BUB1, CCNB2, CCNB1, TOP2A, RRM2, KIF11, KIF20A, NDC80* and *TTK*, were identified via bioinformatic analysis of retinoblastoma tumors that were associated with poor survival outcomes. With the help of liquid chromatography–mass spectrometry, hyperphosphorylated proteins were identified, including stress response proteins H2AFX and SIRT1 and protein kinases BRD4, WNK1 and CDK1*.*

By the use of Methylation Specific Multiplex Ligation Probe Assay hypermethylation in new genes: *T3A, MSH6, CD44, PAX5, GATA5, TP53, VHL* and *GSTP1* has been reported by Livide et al., who also confirmed the already known hypermethylation of *MGMT, RB1* and *CDKN2*. [50]

All the changes observed are summarized in Table 1.

**Table 1:** Genetic, transcriptomic, epigenetic and proteomic changes observed in retinoblastoma

		Mutation type and molecular consequences
Genes	*RB1* [13, 16, 27, 31, 32, 35, 36]	Large spectrum of pathogenic variants, nonsense mutations prevalent; downregulated
	*MYCN* [13, 16, 17, 48]	Amplifications, gains; upregulated
	*BCOR* [16, 48]	Mutations or loss; nonsense variants that result in truncated protein; downregulated
	CREBBP [48, 71, 74, 75]	Nonsense variants that result in truncated protein, downregulated
	*MDM4* [13, 55]	Gains (4‒10 copies); skipping of exons 6 or 9; upregulated
	*KIF14* [13, 51, 52]	Gains (4‒10 copies); upregulated
	*E2F3* [13, 51, 52]	Gains; upregulated
	*DEK* [13, 70–73]	Gains; upregulated
	*OTX2* [16]	Amplification; upregulated
	*RBL2* [16]*, RASSF1A* [65–67]*, MDMX* [3, 13, 52, 70], *CDH11* [13, 52, 70], *nuclear protein 7 (NOL7)* [54]	Loss; downregulated
	*MGMT* [13, 16, 50, 57, 66, 67]	Epigenetic silencing
	*MLH1* [16, 66, 67]	Mutation
	*APOA1, GFAP, RBP3, CRABPs* [3, 53, 54]	Dysregulated
	*CRB1, NUP205, IL8, IL6, MYC, SMAD3, UBE2C, NEK7* [28]*, SKP2, SOX2* [9, 53, 69–73]*, SOX4* [52], *Survivin* [3]*, MDM2* [3]*, CDKN2A (p16INK4A), TFRC* [3, 6, 40, 53, 54, 65]	Overexpression
	*HIST1H4H, RELN, RPTOR, TERT, MSH3, TSC2, ARID1A, CDK4, BRAF, JAK1, ROCK1, ARHGAP9, DRAIC* [74, 75], *CASP8* [66, 67], *LMNB1* [3, 53, 54]	Downregulation
	*ABCA4, DAZAP1, Dab1* [55]	Aberrant splicing
	*TP53, CDH13, GATA5, CHFR, TP73, IGSF4* [50]	Deletions
Chromosomal aberrations	1q, 2p, 6p [49]	Gains
	13q, 16q14 [49]	Losses
lncRNAs	*BANCR, AFAP1-AS1, NEAT1, XIST, ANRIL, PlncRNA-1, HOTAIR, PANDAR, DANCR*, *THOR* [59]	Differentially expressed
miRNAs	miR-216a, miR-217, let-7a, let-7i, let-7f, miR-9, miR-92a, miR-99b, miR-3613, miR486-3p and miR-532 [59–61]	Downregulated
	miR-103, miR-142-5b, miR-106b, miR-143, miR-148b, miR-17, miR-16, miR-183, miR-182, miR-19a, miR-18a, miR-29a, miR-29b, miR-29c, miR-20a, miR-30b, miR-30d, miR-34a, miR-494, miR-378, miR-513, miR-513–1, miR-513–2, miR-518c, miR-96 [59–61]	Upregulated
	miR-320, let-7e, and miR-21 [17]	Dysregulated in plasma of Rb patients
	miR-20a, miR-373, miR-125b, let7a, let-7b, let-7c, miR-25, and miR-18a [60]	Differentially expressed during progression
	miR181b, miR30c-2, miR125a3p, miR497, and miR491-3p [59]	Hypoxia-regulated miRNAs
Epigenetic alterations	*RB1, MGMT, RASSF1A, CASP8, MLH1, CDKN2A/p16INK4A, VHL, LDHA, RUNX3, APC2, PAX5, EZH2, SKY, DNMT1a, DNMT3a, HELLS protein, PLK1, HMGA2,T3A, MSH6, CD44, PAX5, GATA5, TP53, GSTP1, CDK1, BUB1, CCNB2, CCNB1, TOP2A, RRM2, KIF11, KIF20A, NDC80, TTK* [50, 63, 65–67]	Genes with aberrant methylation of promoters
	*H2AFX, SIRT1, BRD4, WNK1, CDK1* [52, 65]	Hyperphosphorylated
Proteomic profiling	IGF2BP1, SOX4, B7H3, chromogranin A, Rac GTPase-activating protein 1, fetuin A, midkine, LRP1 COMP, TGB3, TLN, FLNA, OGN, A1BG, Serpin A1, ORM2, LRG1, CHI3L1, transferrin, TFRC (Transferrin Receptor), alpha-crystallin A, CRABP2 [3, 52, 76–78]	Upregulated
	PEDF, nucleolin (NCL) [76–78]	Downregulated

## Genetic testing and the current treatments for retinoblastoma

The goal of retinoblastoma therapy is to cure the tumor, save the eye and maximize vision, and early detection is of the utmost importance. The good news is that today the survival rates in developed countries are over 95%; however, in lower- and middle-income countries enucleation still remains the treatment of choice and the survival is much lower due to the often treatment failures [3, 79]. For instance, the survival rate and vision retention in one eye in the US is higher than 99% and 90%, respectively [80, 81], while in African patients survival numbers are as low as 40% [82].

The treatment is chosen, among other things, on the basis of the intraocular classification of retinoblastoma (ICRB). Besides big discrepancies in treatment options of retinoblastoma between developing countries and high-income ones, the screening programs for early detection of retinoblastoma in those countries are also rudimentary and not uniform. If diagnosed and treated early, less radical forms of therapy are sufficient to cure retinoblastoma [83]. One of the problems is the late-stage diagnosis, which decreases the chances of saving the ocular globe. Since early-stage diagnosis is crucial many countries introduced the newborn screening for retinoblastoma [84, 85]. The culprits for the variabilities in survival between countries are expensive treatment procedures, and the difference in the availability of specialized ophthalmologists.

In order to screen individuals for their susceptibility to retinoblastoma, the patients together with their relatives must be offered genetic testing for better understanding the nature of inheritance and their constitutional predisposition. In recent years a great number of molecular testing approaches have been developed in order to identify *RB1* gene pathogenic mutation. It is also important to highlight the creation and launch of National Ophthalmic Disease Genotyping and Phenotyping Network (eyeGENE®) by the National Eye Institute (NEI) in 2006. For retinoblastoma specifically, the network encompasses 118 participants who have undergone genetic testing for this pediatric disease (https://eyegene.nih.gov/) [86, 87].

The accuracy of genetic testing is high when DNA can be isolated from tumor DNA after the patients underwent enucleation. Many techniques can be performed with this DNA including Multiplex Ligation-Dependent Probe Amplification (MLPA), Loss of Heterozygosity (LOH), allele-specific PCR, promoter methylation detection, Single Nucleotide Polymorphism (SNP) arrays and finally next-generation sequencing (NGS) followed by Sanger classical sequencing of the recognized regions. For a complete genetic evaluation of the *RB1* gene a multistep assay was developed that includes DNA sequencing to identify mutations within coding exons and immediate flanking intronic regions plus the promoter regions; duplication/deletion analysis and also methylation analysis of the RB1 promoter region. Fortunately, due to the improvement in modern treatment options, fewer enucleations are being performed today thus making the access to tumor DNA for somatic *RB1* analysis restricted. Therefore, genetic testing performed on peripheral blood is becoming more and more important. The accuracy of the peripheral blood tests needs to be high; however, they usually do not test the entire *RB1* locus making them less informative. The regulatory regions of *RB1* or the epigenetic events are also not routinely tested [16]. Genetic testing of the peripheral blood aims to determine the presence of a germline mutation and the absence of pathogenic variants in the germline points to the absence of predisposition. However, the absence of detection of *RB1* pathogenic variant can lead to the conclusion that there is no germline *RB1* pathogenic variant, and therefore no retinoblastoma predisposition, but conventional diagnostic genetic testing does not explore the entire *RB1* locus; thus, an *RB1* germline predisposing pathogenic variant cannot be excluded.

DNA from peripheral blood is tested by genomic hybridization techniques such as chromosomal microarray analyses (CMA). Mutation identification is performed either by traditional Sanger sequencing of the amplified target regions or by SNP arrays. *RB1* custom array-comparative genomic hybridization (aCGH) and NGS methods were recently used to optimize diagnostic molecular testing in retinoblastoma patients [33]. The approach was able to accurately detect point mutations, macrodeletions, duplications and also narrowed down the detection of mosaicism to 1%. Another recent approach that raised the detection frequency of gross deletions and identified genomic abnormalities including germline mosaicism was the use of MLPA and direct sequencing [33, 35].

As mentioned above, the Next-generation Sequencing (NGS) strategies are becoming essential in the management and counseling of patients. Investigations employing targeted NGS using Illumina MiSeq and precision bioinformatic pipelines were also used to identify a spectrum of pathogenic variants in retinoblastoma patients [16, 34, 88].

Notably, direct tissue biopsy is contraindicated for RB due to inaccessibility and the risk of provoking tumor spread [56]. Therefore, the community is pressed to identify trustworthy biomarkers for diagnosis and prognosis. One of novel noninvasive alternative approach to obtain DNA for diagnostic and prognostic purposes, since biopsy is contraindicated, is focusing on cell free DNA (cfDNA) as a surrogate tumor biopsy for retinoblastoma. The source of the tumor genome is cfDNA, and it can be harvested from the anterior segment of the eye—aqueous humor (AH). cfDNA comprises fragments originating from both genomic DNA and circulating tumor DNA (ctDNA) [89]. ctDNA is shown to be representative of the tumor. It consists of small, approximately 200 base pair-long DNA fragments released into the bloodstream by various processes. When collected from the plasma, the ctDNA can be distinguished from genomic DNA based on the presence of cancer-related mutations. In their paper [90], using high-deep next-generation sequencing (NGS) of *RB1* gene have shown that it is possible to detect ctDNA in patients with intraocular unilateral non-hereditary retinoblastoma. By this approach the authors were able to detect 77.8% of previously reported somatic RB1 mutations. However, 15% of patients with unilateral retinoblastoma may still harbor a germline mutation, which indicates the utmost importance of genetic testing of the peripheral blood.

ctDNA can be found both in aqueous humor (AH) and plasma and represents an excellent biomarker for retinoblastoma that can also be used at later instances during the follow-up of the patients [91–93].

## Conclusions

Retinoblastoma is one of the first tumors that demonstrated the tumor suppressor role of *RB1* gene. In spite of extensive modern mutational investigations, the *RB1* gene remains the master gene responsible for this disease. Comprehending retinoblastoma molecular genetics is crucial for diagnostic and prognostic purposes. However, the molecular diagnosis of RB is a complex process in which high-throughput technologies can greatly contribute. It has been shown that besides *RB1* mutations additional genes are also involved, primarily *MYCN,* whose amplifications are found in patients without *RB1* inactivation. Recurrent mutations in *BCOR* and *CREBBP* genes have been described in a small percentage of tumors, too. Furthermore, epigenetic changes contribute to the progression of retinoblastoma as well indicating important role of pRB protein in chromatin remodeling. These findings led to a proposal of categorization of retinoblastoma subtypes. Another novel approach that shows potential in improving therapeutic solutions and outcomes for affected children is the use of cfDNA and tfDNA. The tumor genome is accessible through tfDNA in the anterior segment of the eye which is a very useful source of DNA for diagnostics and can help predict which eyes can be salvaged. Future investigation need to identify credible biomarkers and molecular targets for improving diagnostic and treatment options.

## Abbreviations

RB: Retinoblastoma
RB1: Retinoblastoma 1 gene
pRb: Retinoblastoma protein
BCOR: BCL6 Corepressor gene
CREBBP: CREB-binding protein gene
DNA: Deoxyribonucleic acid
ICRB: Intraocular Classification of Retinoblastoma
IIRC: International Intraocular Retinoblastoma Classification
cTNMH: Clinical tumor, lymph nodes, systemic metastasis and hereditary
AJCC: American Joint Committee on Cancer
IRSS: International Retinoblastoma Staging System
RPC: Retinal progenitor cell
SNV: Single nucleotide variants
MLPA: Multiplex-ligation dependent probe amplification
KDa: Kilodaltons
RBN: Retinoblastoma protein N-terminal domain
RBP: Retinoblastoma protein pocket
RBC: Retinoblastoma protein C-terminal domain
HDACs: Histone deacetylases
CDK: Cyclin-dependent kinase
CKIs: Cyclin-dependent kinase inhibitors
CIN: Cervical intra-epithelial neoplasia
CDH11: Cadherin-1
APOA1: Apolipoprotein A1
CDKN2A: Cyclin Dependent Kinase Inhibitor 2A
CRABPs: Cellular Retinoic Acid Binding Proteins
LMNB1: Lamin B1
TFRC: Transferrin Receptor
NGS: Next-generation sequencing
miRNAs: MicroRNAs
lncRNA: Long non-coding RNA
EZH2: Zeste 2 polycomb gene
NEI: National Eye Institute
LOH: Loss of Heterozygosity
PCR: Polymerase chain reaction
SNP: Single nucleotide polymorphisms
CMA: Chromosomal microarray analyses
aCGH: Array-comparative genomic hybridization
cfDNA: Cell free DNA
AH: Aqueous humor
ctDNA: Circulating tumor DNA
